# A Pharmacometabolomic Approach to Predict Response to Metformin in Early-Phase Type 2 Diabetes Mellitus Patients

**DOI:** 10.3390/molecules23071579

**Published:** 2018-06-29

**Authors:** Jeong-Eun Park, Gui-Hwa Jeong, In-Kyu Lee, Young-Ran Yoon, Kwang-Hyeon Liu, Namyi Gu, Kwang-Hee Shin

**Affiliations:** 1 College of Pharmacy, Research Institute of Pharmaceutical Sciences, Kyungpook National University, Daegu 41566, Korea; knu_park@knu.ac.kr (J.-E.P.); dstlkh@gmail.com (K.-H.L.); 2 Department of Endocrinology, Changwon Fatima Hospital, Changwon 51394, Korea; hyunnii-j@hanmail.net; 3 Department of Endocrinology, Kyungpook National University Hospital, Daegu 41944, Korea; leei@knu.ac.kr; 4 Department of Biomedical Science, BK21 Plus KNU Bio-Medical Convergence Program for Creative Talent, Cell and Matrix Research Institute and Clinical Trial Center, Kyungpook National University Graduate School and Hospital, Daegu 41944, Korea; yry@knu.ac.kr; 5 Department of Clinical Pharmacology and Therapeutics, Clinical Trial Center, Dongguk University College of Medicine and Ilsan Hospital, Goyang 10326, Korea; namyi.gu@gmail.com

**Keywords:** metformin, type 2 diabetes mellitus, inter-individual variability, pharmacometabolomics

## Abstract

Metformin is a first-line medication for type 2 diabetes mellitus (T2DM). Based on its universal use, the consideration of inter-individual variability and development of predictive biomarkers are clinically significant. We aimed to identify endogenous markers of metformin responses using a pharmacometabolomic approach. Twenty-nine patients with early-phase T2DM were enrolled and orally administered metformin daily for 6 months. A total of 22 subjects were included in the final analysis. Patients were defined as responders or non-responders based on changes in their glycated haemoglobin A1c (HbA1c) from baseline, over 3 months. Urine metabolites at baseline, as well as at the 3 and 6 month follow-ups after the start of treatment were analysed using gas chromatography-mass spectrometry and evaluated with multivariate analyses. Metabolites distinguishable between the two response groups were obtained at baseline, as well as at the 3 and 6 month follow-ups, and significantly different metabolites were listed as markers of metformin response. Among the identified metabolites, citric acid, myoinositol, and hippuric acid levels showed particularly significant differences between the non-responder and responder groups. We thus identified different metabolite profiles in the two groups of T2DM patients after metformin administration, using pharmacometabolomics. These results might facilitate a better understanding and prediction of metformin response and its variability in individual patients.

## 1. Introduction

Type 2 diabetes mellitus (T2DM) is considered a global pandemic, and its incidence is steadily increasing [1]. The worldwide prevalence of patients with T2DM is expected to increase from 2.8% in the 2000s to 4.4% in 30 years [2]. Furthermore, its prevalence is particularly high in China and India, which are the two most populous countries in Asia and the world [2]. The prevalence of T2DM in Korea has increased 3-fold over the past 30 years, and the mortality rate of diabetes is the fourth highest in this country [3,4]. Impaired glucose tolerance and fasting glycaemia due to irregular carbohydrate metabolism have been identified as T2DM risk factors [5].

Metformin, a biguanide antihyperglycaemic agent, is frequently used as the first-line therapeutic agent for T2DM, and has been shown to have numerous benefits, such as weight loss, lowering of lipid levels, and decreasing diabetes-related mortality [6,7]. The major antihyperglycaemic actions of metformin involve reducing hepatic glucose production during gluconeogenesis [8] and increasing the insulin sensitivity of muscles [9]. However, large inter-individual variations have been reported in the responses of patients administered metformin. In previous studies, metformin was considered to have failed to control glucose levels in approximately 50% of patients, among whom the fasting plasma glucose concentration was more than 140 mg/dL or the glycosylated haemoglobin A1c (HbA1c) was more than 7% even after metformin therapy [10,11]. Genetic polymorphisms or other external or internal factors may cause patient unresponsiveness to metformin treatment [12]. For example, a lower effectiveness was observed in diabetic patients with a minor C allele at the single nucleotide polymorphism (SNP) rs622342 of the gene *SLC22A1*, and this genetic variant was associated with weaker glucose-lowering effects after metformin treatment [12].

Pharmacometabolomics is a useful approach for predicting the efficacy or toxicity of a particular drug intervention or measuring variations in metabolites due to environmental factors or diseases [13]. Among the several omics tools, metabolomics addresses the end points of biochemical reactions, which are well-reflected in biological changes, and it covers overall biological changes from genomic- to proteomic levels [14]. Pharmacometabolomics is a global biochemical approach that makes a significant contribution to pharmacokinetics and pharmacogenetics, and provides insights into the mechanisms involved in drug responses, including at the level of genetics [13,15]. Recently, numerous studies on the correlation between the risk of type 2 diabetes mellitus progression and certain metabolites or changes in metabolites after antidiabetic drugs treatment have been performed [16,17,18]. 

Several endogenous metabolites were associated with the risk of developing T2DM in previous studies. For example, Rotroff et al. [16] showed that 12 metabolites were significantly associated with metformin-induced changes in glucose in the plasma of non-diabetic participants when they were administered the oral glucose tolerance test. Among these metabolites, the 2-hydroxybutanoic acid level was considered to be a potential predictor of metformin response [16].

The aim of this study was to identify urinary markers for inter-individual variations in the metformin response of patients newly diagnosed with T2DM and administered metformin for 6 months using a global pharmacometabolomic approach with gas chromatography-mass spectrometry (GC/MS).

## 2. Results

### 2.1. Subject Characteristics

Metformin was orally administered to 22 patients with T2DM daily for 6 months. Clinical variable measurement and urine collection were performed before metformin administration (at baseline), and also 3 and 6 months after the start of the metformin treatment, of which the actual mean ± SD sampling periods were 93.4 ± 3.77 and 187 ± 5.12 days after baseline, respectively. The differences in the clinical variables of the two phenotypes at the baseline, as well as the 3 and 6 month follow-ups after metformin administration began are shown in Table 1. At baseline, the body mass index (BMI) in the responder and non-responder groups was similar, 27.1 ± 1.96 and 25.5 ± 2.91, respectively. Moreover, the physical activity and dietary control levels were not different between the two groups. Additionally, biochemical data for glucose and insulin concentration differences between the two phenotypes were not observed. However, the HbA1c levels were approximately 0.13-fold lower in the non-response group than in the response group at baseline. The changes in HbA1c after 3 months and 6 months of treatment were −13.4% (*p* < 0.0001) and −12.8% (*p* = 0.011), respectively, in the response group, but the differences were not significance in the non-response group (0.05% [*p* = 0.975] and −1.96% [*p* = 0.249] after 3 and 6 months of metformin treatment, respectively). Before the treatment, there were no clinical parameters that differed between the two groups, except for the HbA1c.

### 2.2. Multivariate Analyses

**Figure 1:**
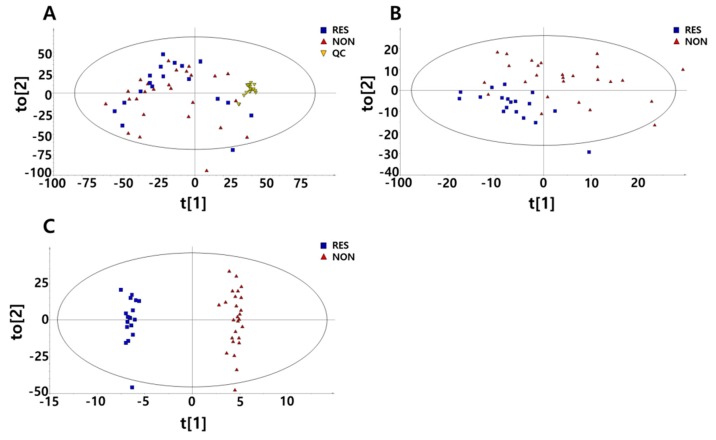
Multivariate analyses derived from untargeted metabolic profiling using gas chromatography/mass chromatography (GC/MS). Data are shown for 9 responders and 13 non-responders before and after 3 months of metformin treatment. (**A**) Principal component analysis (PCA) with 18 additional pooled urine samples added for quality control (QC). (**B**) Partial least squares-discriminant analysis (PLS-DA) score plots divided into responders and non-responders to metformin. (**C**) orthogonal PLS (OPLS)-DA score plots divided into responders and non-responders to metformin.

To identify the metabolic variations in the mass spectra of both study groups, multivariate methods were used to analyse the data of responder and non-responder patients. The data consisted of the metabolite levels at baseline and 3 months after treatment (Figure 1). The principal component analysis (PCA) plot revealed a slight difference between the metformin responder and non-responder groups. When all the pooled urine was used as quality control samples, the plots were confirmed to be well gathered, which indicates that there was no inconsistency in the mechanical analysis or error in the method development (Figure 1A). To obtain a successful separation that reflects metabolite variables, a partial least squares-discriminant analysis (PLS-DA) model that provided obvious proof of the difference between the responders and non-responders was performed (Figure 1B). An improved PLS-DA model was constructed using orthogonal PLS (OPLS)-DA analysis to clearly visualize the difference between the two groups (Figure 1C).

### 2.3. GC/MS of Untargeted Metabolomics in Urine

The differential metabolites tested had a variable importance for the projection (VIP) value that was 1.0 or more in the PLS-DA model. A total of 470 variables that were prominent discriminators between the responder and non-responder groups were found. Of these, 11 candidates (citric acid, p-cresol glucuronide, uric acid, gluconic acid, myoinositol, pseudouridine, p-hydroxyphenylacetic acid, D-threo-isocitric acid, hippuric acid, hypoxanthine, and 3-(3-hydroxyphenyl)-3-hydroxypropanoic acid) were obtained, based on the peak detection time and mass spectra, under the criteria that the similarity was 80% or more to the values in the MassHunter library. Finally, citric acid, myoinositol, pseudouridine, p-hydroxyphenylacetic acid, hippuric acid, and hypoxanthine were listed as candidate metabolites that could potentially reflect the responsiveness for metformin.

**Figure 2:**
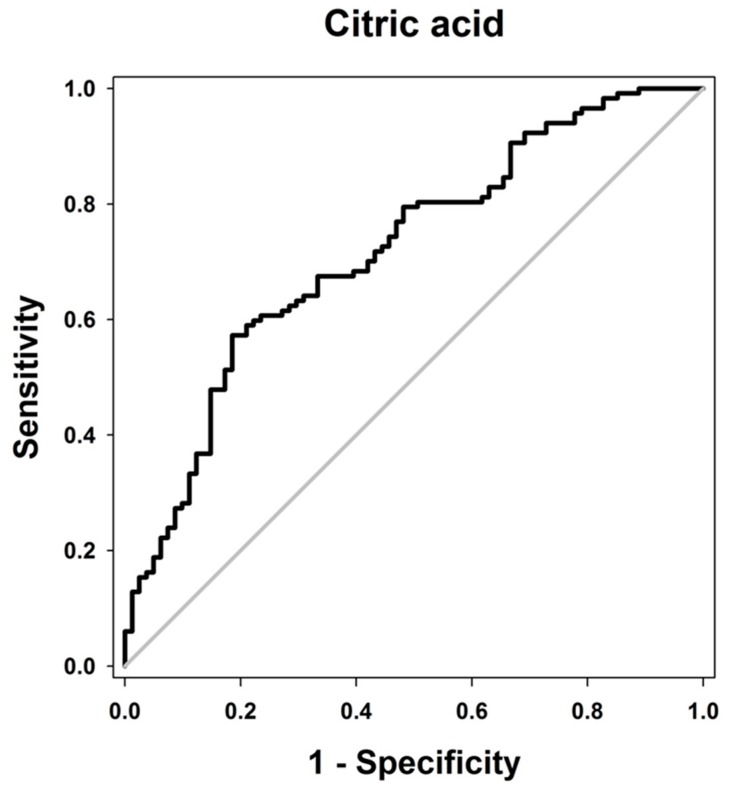
Receiver operating characteristic (ROC) curve illustrating the diagnostic potential of significant metabolites. The ROC curve of citric acid relative intensity before metformin administration is plotted. The area under the ROC curve was 0.719 (*p* < 0.0001).

Of the six compounds identified above, the relative intensity values of citric acid, myoinositol, and hippuric acid at baseline showed significant differences between the two phenotypic groups (Table 2). Citric acid and hippuric acid showed lower relative intensity levels in the non-responder group than they did in the responder group, with baseline differences of −54.6% (*p* < 0.0001) and −72.3% (*p* = 0.018), respectively (Table 2). In contrast, the peak relative intensity of myoinositol at baseline was 18.1% higher in the non-responder group than that in the responder group (*p* = 0.049, Table 2). Furthermore, the area under the curve (AUC) of the receiver operating characteristic (ROC) curve for citric acid at baseline showed a significant value of 0.719 (*p* < 0.0001), suggesting it could be applied to predict the phenotypes (Figure 2).

The changes in the six metabolites after 3 and 6 months of treatment are presented separately for the two phenotype groups, in Table 3. Citric acid increased significantly in the non-responders after 3 and 6 months of treatment (*p* = 0.001 and *p* < 0.0001, respectively), and simultaneously increased with the duration of metformin administration. However, in the responder group, the relative intensity change of this metabolite was not different after treatment compared with that at baseline. However, myoinositol showed a significant decrease in both phenotype groups after 3 and 6 months compared to baseline (*p* < 0.0001 in all groups). In the non-responder group, there was a tendency for this to decrease less than that in the responder group. Except for the above two metabolites, the changes in the remaining four metabolites did not show any significant differences in both the responder and the non-responder group compared to the baseline values.

## 3. Discussion

Using global metabolomics for urine samples from early-phase T2DM patients, metabolite markers to predict the metformin response within 6 months were identified. Three metabolites, citric acid (54.6% lower in non-responders), myoinositol (18.1% higher in non-responders), and hippuric acid (72.3% lower in non-responders), were significantly different between the responders and non-responders at baseline, which might have the potential to be diagnostic biomarkers that could predict the metformin responses in T2DM patients.

Citric acid and myoinositol were previously reported to be indicators of the risk of diabetes development [19,20]. In particular, citric acid was reported to play an essential role in regulating glucose-induced insulin release at the centre of the tricarboxylic acid (TCA) cycle, and the citric acid levels of T2DM patients were 0.43-fold higher than that in normal participants, but slightly decreased after metformin treatment [19]. Metformin inhibits the activity of the citric acid cycle [21], resulting in a lower concentration of citric acid. In the present study, the citric acid level in the non-response group was 54.6% lower than that of responders at baseline, and citric acid levels significantly increased at the 3 (*p* = 0.001) and 6 month follow-ups (*p* < 0.0001), after the start of metformin treatment. Therefore, patients with low concentrations of citric acid in the urine could prove to be relatively less affected by the metformin treatment compared with those with higher levels of citric acid.

The urinary excretion of myoinositol over 24 h was shown to be increased in diabetic subjects [20], which could make it a good indicator of T2DM [22]. The high concentration of myoinositol in the urine of diabetic patients is due to its competitive inhibition with glucose in renal tubular transport [22], and thus, the urine concentration of myoinositol is expected to decrease after treatment with antidiabetic drugs. In this study, myoinositol had a higher level of 18.13% in the non-responder group, and decreased in both phenotypes after metformin monotherapy. In addition, the group with the lower level of myoinositol at baseline (responders) also had a smaller decrease in this metabolite after both 3 and 6 months of treatment (Table 3). Therefore, a high concentration of myoinositol in the urine may be a biological marker of both T2DM and the non-response of patients to metformin treatment.

The hippuric acid level was previously reported to decrease in T2DM patients with impaired glucose tolerance (IGT), and was increased again after treatment with anti-T2DM medication [23]. Additionally, the reduction of hippuric acid levels derived from gut microflora metabolism breakdown was associated with pre-diabetic individuals [17,24]. In this study, the level of hippuric acid in the non-response group was 72.3% lower than that in the responder group at baseline, which could explain the non-responsiveness of metformin, as well as the co-occurrence of T2DM with low concentrations of hippuric acid.

**Figure 3:**
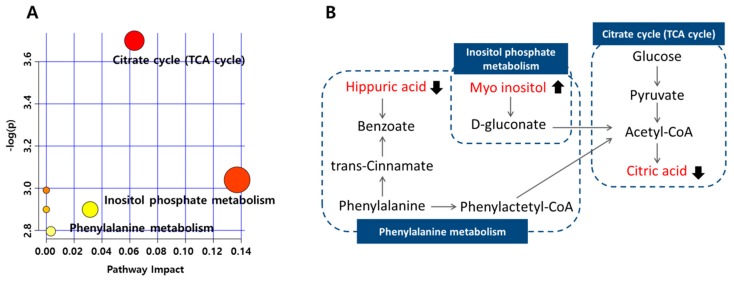
Pathway analysis of metabolites associated with metformin non-response at baseline, according to the MetaboAnalyst (MetPA). (**A**) Metabolome pathways of the citric acid, myoinositol, and hippuric acid; the larger the value of -log(p), the more red points are represented in the plot (based on p-values from pathway enrichment analysis), and the node radius was based on their impact value (impact values from pathway topology analysis). (**B**) Symbols: filled arrow indicates significant difference in non-responder groups at baseline.

From the pathway analysis conducted, glucose metabolism was shown to have a prominent role in this biochemical reaction (Figure 3). Furthermore, hippuric acid and myoinositol metabolism were closely related to the citric acid cycle. The p-hydroxyphenylacetic acid, hypoxanthine, and pseudouridine levels did not show any significant difference between the responders and non-responders at baseline. p-hydroxyphenylacetic acid occurs in the metabolism of phenylalanine. Previous studies have reported that the phenylalanine level is consistently associated with the risk of developing type 2 diabetes mellitus [25], and thus its metabolite, p-hydroxyphenylacetic acid, could also be associated with T2DM. The levels of xanthine and hypoxanthine in patients with T2DM were shown to be increased compared with those of the participants without diabetes [24,26]. In addition, pseudouridine has also been studied and found to be positively associated with the progression of type 2 diabetes by inhibiting glucose utilization [27]; moreover, it was reported to have a significant relationship with the change in HbA1c levels in the metformin-treated patients [28]. All three metabolites mentioned above were associated with the progression of T2DM, and appeared to differ in the two phenotypes before metformin administration, but these differences were not statistically significant. Thus, it was deemed that these metabolites may be connected with mechanisms of metformin non-response, but there was not enough evidence to conclude that these were suitable for use as markers to predict metformin response.

This study has several limitations. First, the study was performed on a small number of patients, so the ability to extrapolate from the results might be limited. Additional studies with a large number of patients should be conducted for a more comprehensive assessment. Second, the dose of metformin was not completely controlled. All the non-responders were administered 500 mg of metformin, but two of the nine responders were administered 1000 mg. The effect of dose on the HbA1c changes did not show a significant difference between the two dose groups. Moreover, a previous meta-analysis of the effect of oral antidiabetic agents on HbA1c levels suggested a lack of evidence for HbA1 reduction with an increase in the dose of metformin [29]. Next, five metabolites out of 11 candidates were not identified with the current analytical method. Despite these limitations, the final metabolites that were derived from our full screening of global pharmacometabolomics were significantly correlated with the control of T2DM with metformin.

## 4. Materials and Methods 

### 4.1. Study Design and Subjects

**Figure 4:**
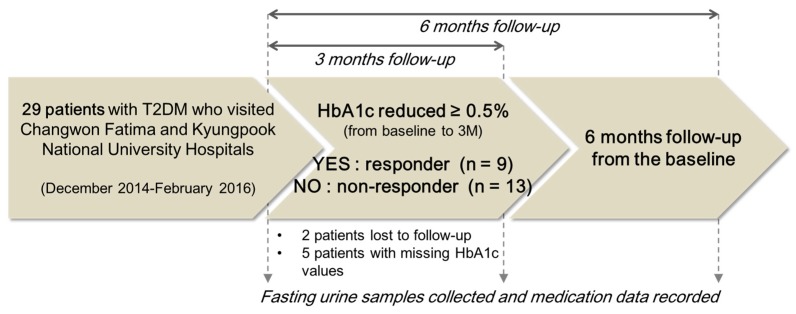
Characteristics of the study subjects.

A total of 29 patients in the early phase of T2DM were enrolled in this study from two hospitals, the Korea Changwon Fatima and Kyungpook National University Hospitals, Korea. The patients treated with metformin at 500 or 1000 mg per day for 6 months were enrolled in the study. Before participating in the study, the patients provided written consent. Twenty-seven patients completed the 6 month metformin therapy, and two patients were lost during the follow-up. Among the 27 patients, five subjects were excluded because their HbA1c records were missed. Per-protocol analysis was conducted to study the final 22 subjects (10 men and 12 women, with an average age of 53 years). Patients were classified into two phenotypic groups according to their metformin response, as follows: (1) the responder group, in which HbA1c levels decreased 0.5% or more after 3 months compared with the pre-treatment (baseline) levels; and (2) the non-responder group, in which HbA1c levels decreased less than 0.5% after 3 months compared with baseline (Figure 4) [30,31]. The patients’ anthropometric parameters, biochemical data, and medication information were recorded at baseline and at the follow-ups 3 and 6 months after the start of metformin treatment. In addition to data on the clinical variables of the patients, conditions that could affect the metformin response were recorded from patients’ self-reporting. Physical activity levels were documented as hours of activity per week, and the dietary control of food intake was recorded as scores from 1 to 10 (Table 1). Fasting urine samples were obtained for endogenous metabolite measurements at these three measurement points. The protocol of this study was approved by the Institutional Review Boards (IRBs) of the Changwon Fatima and Kyungpook National University Hospitals, Korea (IRB Nos. 14-15-01 and 2014-10-016-001, respectively). All procedures were conducted in accordance with the principles of the Declaration of Helsinki (59th World Medical Association General Assembly, Seoul, Korea, October 2008) and Good Clinical Practice (GCP) guidelines.

### 4.2. Sample Preparation and MS Analysis

Urine samples were collected under fasting conditions to avoid variations caused by food effects. Urine samples were stored at −80 °C, and were then thawed, slightly mixed, and transferred in 100-µL aliquots to 1.5 mL polypropylene tubes. Following the addition of 40 µL of urease (10 mg/mL), the samples were incubated for 30 min at 37 °C to remove urea. Next, 400 µL of cold methanol and 10 µL of heptadecanoic acid (10 mg/mL), which was used as the internal standard, were added, mixed by vortexing, and centrifuged at 16,168× *g* for 10 min at 4 °C. A 200-µL aliquot of the supernatant was evaporated to dryness using a speed vacuum at 45 °C for 3 h. After the addition of 50 µL of methoxamine (MOX) reagent (Thermo Scientific, Waltham, MA, USA), the mixture was vortexed for 5 min and agitated for 90 min at 30 °C, using a shaking water bath. Next, 50 µL of *N*-methyl-*N*-(trimethylsilyl)trifluoroacetamide (MSTFA) with 1% (*v*/*v*) trimethylchlorosilane (TMCS, MSTFA + 1% TMCS, Thermo Scientific, USA) was added, and the tubes were vortexed for 5 min and then centrifuged at 16,168× *g* for 10 min at 4 °C. Finally, the derivatized samples were transferred to GC/MS vials, and the injection volume was 1 µL into the GC/MS system. An Agilent 7890A gas chromatograph coupled to a 5975C mass spectrometer (both from Agilent Technologies, Palo Alto, CA, USA) was used for the analysis of untargeted metabolomics. The components were separated using an Agilent (Agilent Technologies, Palo Alto, CA, USA) 30 m × 0.25 mm × 0.25 mm HP-5MS column. Briefly, each 1 µL sample was loaded into the GC/MS using helium as the carrier gas, at a flow rate of 1.2 mL/min in split injection mode, with a split ratio of 1:5. The initial oven temperature was 70 °C for 0.1 min, which was then increased by 5 °C/min up to 310 °C, where it was maintained for 2 min. The MS data were analysed in the full-scan mode after a 13 min solvent delay time with a mass to charge ratio (*m*/*z*) scan range of 50–800. To validate the overall analysis procedure, quality control (QC) was assessed using a pooled urine sample. All derived endogenous metabolites’ intensities were normalized using the intensity of the internal standard (heptadecanoic acid). The analytical process was demonstrated to have been appropriate through analysis of the pooled urine QC identity.

### 4.3. Data Processing for Metabolomics

The mass data were analysed using the Agilent MassHunter search algorithm (Agilent Technologies, Palo Alto, CA, USA) for comparing spectrum extraction, fragmentation patterns, and integration of the peak areas of compounds. The data from the MS scan were normalized before the data alignment with R version 3.3.1 (http://cran.r-project.org/) [32]. In addition, the normalized data were matched and aligned using the Xcms package in R software. The urine metabolites were evaluated with multivariate statistical analyses using the SIMCA 13 software (Umetrics, Umea, Sweden). PCA and a PLS-DA provided the same weights of variables when illustrated using reasonable clustering [33]. The PCA score plot was used to determine the differences or similarities between the groups by checking for visible segregation into clusters on data plots. The PLS-DA based on PCA information was used as a supervised classification method [34]. More specifically, the R^2^ value was used to determine the goodness-of-fit of the model, and the Q^2^ value was used to determine the accuracy of prediction with another data set. The R^2^ and Q^2^ ranged between 0 and 1, with 1 representing perfect fit and predictive ability, respectively [35]. The pathways of the metabolites identified in this study were illustrated using the MetaboAnalyst 4.0 program, based on metabolic pathways in the high-quality Kyoto Encyclopaedia of Genes and Genomes (KEGG) [36,37].

### 4.4. Identification of Urine Metabolites

A compound analysis was performed to identify the candidate metabolites of citric acid, uric acid, gluconic acid, myoinositol, pseudouridine, p-hydroxyphenylacetic acid, hippuric acid, and hypoxanthine, which were commercially available among the eleven metabolites derived from the MassHunter library. The standards of these candidates were purchased from Sigma-Aldrich (St. Louis, MO, USA), except for peudouridine, which was obtained from Carbosynth, Compton, Berkshire, UK. All of the compounds were used at an adjusted concentration of 0.5 mg/mL and centrifuged at 16,168× *g* for 10 min at 4 °C. After drying 30 µL of the supernatant, the process was the same as the pre-treatment of the urine samples, including use of MOX reagent and MSTFA + 1% TMCS. Then, 1 µL of the sample was injected into GC/MS under the same condition as in the urine sample analysis. The Human Metabolome Database (HMDB, http://www.hmdb.ca/) and MassHunter library were used to search the peak detection times and the mass spectra of the metabolite molecules. Finally, citric acid (detected at 23.28 min), myoinositol (detected at 28.53 min), pseudouridine (detected at 32.82 min), p-hydroxyphenylacetic acid (detected at 19.04 min), hippuric acid (detected at 23.22 min), and hypoxanthine (detected at 22.65 min) were identified. As peaks were not observed for uric acid and gluconic acid under the experimental conditions, these metabolites were excluded from the final analyses of metabolite markers.

### 4.5. Statistical Analyses

The clinical characteristics data were expressed as means ± standard deviations (SDs), and were evaluated using the Student’s *t*-test or the Wilcoxon signed-rank test, using SPSS statistical software (IBM SPSS Statistics 22, Chicago, IL, USA). All metabolite measurements were expressed as differences between the values at baseline and the two measurement points (3 month and 6 month follow-ups), and were presented as means ± SDs of the differences. Statistical analyses of the normalized data were performed using the Student’s *t*-test and Wilcoxon signed-rank test. The area under the ROC curve was calculated to demonstrate whether there was a correlation between the response or non-response of patients to metformin and specific metabolite levels. The ROC consisted of an X line, called 1-specificity (false positive rate), versus a Y line, or sensitivity (true positive rate), which are associated with the specific disease or response to treatment [38]. The AUC of the ROC was evaluated to obtain a powerful confirmation of the differential metabolites between the responder and non-responder groups. The ROCs were plotted using SigmaPlot software version 1.0 (Systat Software Inc., Richmond, CA, USA). If the ROC graph was concentrated towards the left and top vertex, then the AUC is close to 1, which means that the predictive model has high true positive and low false positive rate. According to the AUC, the test is judged to have moderate accuracy when the AUC is more than 0.7, and less accuracy when it is less than 0.7 [39].

## 5. Conclusions

The current study demonstrated that there were metabolic differences in the metformin responder and non-responder groups, even at baseline. Differential metabolites in the urine, such as citric acid, hippuric acid, and myoinositol, which were significantly different at baseline, might play a major role in distinguishing the two phenotypes. Lower baseline levels of citric acid and hippuric acid and higher baseline levels of myoinositol in patients with T2DM were related to their subsequent non-response to metformin. These results suggested that these metabolites could be used as biomarkers to predict potential metformin responses.

## Figures and Tables

**Table 1:** Characteristics of responder and non-responder groups at baseline, and after 3 months (Post 3M) and 6 months (Post 6M) of metformin administration (*n* = 22).

	Baseline			Post 3M			Post 6M		
	Responder (*n* = 9)	Non-responder (*n* = 13)	*p*	Responder (*n* = 9)	Non-responder (*n* = 13)	*p*	Responder (*n* = 9)	Non-responder (*n* = 13)	*p*
Age, y	54.3 ± 8.90	52.2 ± 7.75	^a^ 0.574	-			-		
Males, %	22.2	61.5	^a^ 0.068	-			-		
Height, cm	159 ± 5.05	164 ± 9.09	^a^ 0.102	-			-		
Weight, kg	68.4 ± 5.73	68.3 ± 10.7	^a^ 0.978	66.3 ± 5.12	67.4 ± 10.5	^a^ 0.754	66.2 ± 4.91	67.3 ± 10.2	^a^ 0.750
Body Mass Index, kg/m^2^	27.1 ± 1.96	25.5 ± 2.91	^a^ 0.119	26.3 ± 1.68	25.1 ± 2.89	^a^ 0.227	26.3 ± 1.61	25.1 ± 2.66	^a^ 0.200
Waist Circumference, cm	87.2 ± 3.82	87.4 ± 7.36	^a^ 0.933	88.7 ± 5.38	86.7 ± 7.11	^a^ 0.499	88.7 ± 5.20	86.8 ± 7.62	^a^ 0.553
Systolic Blood Pressure, mmHg	123 ± 15.5	129 ± 11.0	^a^ 0.332	117 ± 14.4	128 ± 16.6	^a^ 0.132	126 ± 10.8	127 ± 11.2	^a^ 0.802
Diastolic Blood Pressure, mmHg	72.8 ± 15.3	78.9 ± 5.24	^a^ 0.276	68.4 ± 6.80	77.4 ± 8.31	^a^ 0.012 *	72.2 ± 10.5	78.1 ± 6.59	^a^ 0.163
Heart Rate/Pulse Rate, beats/min	76.4 ± 8.75	80.1 ± 10.3	^a^ 0.722	76.3 ± 14.0	79.4 ± 12.1	^b^ 0.575	74.8 ± 12.4	76.8 ± 12.2	^a^ 0.710
Physical Activity Per Week	2.07 ± 2.07	2.23 ± 2.64	^b^ 1.000	3.94 ± 3.08	3.96 ± 2.84	^b^ 0.893	2.44 ± 2.60	4.04 ± 2.97	^b^ 0.293
Dietary Control	6.38 ± 1.85	4.90 ± 1.52	^b^ 0.091	7.38 ± 1.51	6.14 ± 2.24	^a^ 0.168	6.88 ± 1.73	5.95 ± 2.15	^a^ 0.316
Glucose, mg/dL	142 ± 31.5	135 ± 21.2	^a^ 0.555	121 ± 20.5	135 ± 14.5	^a^ 0.112	120 ± 19.6	130 ± 19.2	^a^ 0.235
Insulin, µIV/mL	10.8 ± 6.90	8.82 ± 4.81	^b^ 0.594	8.24 ± 3.16	7.96 ± 3.52	^a^ 0.852	10.4 ± 3.73	9.67 ± 4.85	^a^ 0.698
Hb1Ac, %	7.77 ± 1.14	6.78 ± 0.47	^b^ 0.028 *	6.73 ± 0.85	6.78 ± 0.50	^b^ 0.767	6.78 ± 0.53	6.64 ± 0.57	^a^ 0.585

Data are means ± standard deviations. *p*-values are shown at the time of measurement of each of the three clinical variables of the difference between the response group and the non-response group. ^a^ Unpaired *t*-test; ^b^ Wilcoxon signed-rank test; * *p*-value ≤0.05.

**Table 2:** Different relative intensity levels in responder and non-responder groups at baseline.

Metabolites	Responder	Non-Responder	% Difference	*p*	Similarity (%)	RT (minute)
**Citric Acid**	1.33 ± 1.50	0.61 ± 0.57	−54.6	<0.0001 **	95.4	23.27
**Myoinositol**	1.99 ± 3.11	2.35 ± 3.63	18.1	0.049 *	85.9	28.48
**Pseudouridine**	4.04 ± 11.7	0.10 ± 0.09	−97.5	0.139	80.3	32.80
**p-hydroxyphenylacetic Acid**	0.30 ± 0.26	0.23 ± 0.26	−24.1	0.719	91.1	19.02
**Hippuric Acid**	10.9 ± 48.4	5.78 ± 8.75	−72.3	0.018 *	97.4	23.22
**Hypoxanthine**	1.72 ± 2.96	1.86 ± 5.16	8.25	0.859	95.4	22.68

Data are means ± standard deviations. *p*-values were calculated by the Wilcoxon signed-rank test; * *p*-value ≤ 0.05; ** *p*-value ≤ 0.001. Percentage difference between responders and non-responders at baseline was calculated as follows: (Non-responder − Responder)/Responder × 100%. Similarity means a value obtained by comparing the urine sample spectrum and the MassHunter library. RT is retention time from the urine sample.

**Table 3:** Relative intensity levels in responder and non-responder groups after 3 (3M) and 6 months (6M) of metformin treatment compared to the baseline.

Metabolites	Responder (3M)	*p*	Non-Responder (3M)	*p*	Responder (6M)	*p*	Non-Responder (6M)	*p*
**Citric Acid**	−0.07 ± 1.58	0.060	0.27 ± 0.66	0.001 **	0.04 ± 2.48	0.112	0.53 ± 1.89	<0.0001 **
**Myoinositol**	−1.25 ± 2.57	<0.0001 **	−0.70 ± 1.89	<0.0001 **	−1.30 ± 2.84	<0.0001 **	−1.11 ± 2.66	<0.0001 **
**Pseudouridine**	−3.17 ± 9.56	0.767	0.01 ± 1.16	0.807	−2.90 ± 8.58	0.214	0.08 ± 0.19	0.173
**p-hydroxyphenylacetic Acid**	0.02 ± 0.32	0.486	−0.08 ± 0.28	0.387	−0.07 ± 0.36	0.136	−0.07 ± 0.25	0.084
**Hippuric Acid**	−2.68 ± 51.5	0.199	6.52 ± 18.6	0.304	7.19 ± 83.3	0.845	15.1 ± 64.6	0.200
**Hypoxanthine**	1.00 ± 5.79	0.594	−0.81 ± 4.77	0.552	−1.29 ± 2.89	0.214	−1.03 ± 4.52	0.807

Relative intensity levels indicate changes after 3 and 6 months of metformin treatment in responders and non-responders as follows: (post dose – baseline). Data are means ± standard deviations. The Wilcoxon signed-rank test was used to determine the significance level of the degree of change relative to the baseline as the *p*-value; ** *p*-value ≤ 0.001.
